# Prescription medicines, over-the-counter medicines and complementary and alternative medicines use: a comparison between baby boomers and older South Australians

**DOI:** 10.3934/publichealth.2019.4.380

**Published:** 2019-10-14

**Authors:** Bee Leng Per, Anne W Taylor, Tiffany K Gill

**Affiliations:** 1 SA Pharmacy, Central Adelaide Local Health Network, Royal Adelaide Hospital, Adelaide, SA, Australia; 2 Adelaide Medical School, Faculty of Health and Medical Sciences, The University of Adelaide, Adelaide, SA, Australia

**Keywords:** baby boomers, older people, polypharmacy, medicines use

## Abstract

**Objective:**

This study examines the difference in medication use between baby boomers (born between 1946–1965) and older people (born before 1946) to determine the proportion of people combining over-the-counter (OTC) medicines and complementary and alternative medicines (CAM) use with prescription medicine use.

**Design:**

A clustered, multistage, systematic, random, self-weighting area sample was obtained and a face-to-face interview was conducted to examine the difference in use in prescription medicines, OTC, and CAM and factors associated with the use between baby boomers and older people.

**Setting:**

South Australia.

**Participants:**

Respondents aged 15 years and over participated in surveys conducted in autumn (March to May) of 2004 (n = 3015) and 2008 (n = 3,034) in which all respondents were asked to list their current medications. This study focuses on those participants whose age was in the range defined by baby boomers and older people.

**Main outcome measures:**

Proportion in each age group taking prescription medicine, OTC medicine, and CAM were determined. Multivariable logistic regression analyses were performed to investigate the relationships between medication use and demographic variables.

**Results:**

The results showed that older people were not only the higher users of prescriptions medicines but also OTC medicines and CAM. Gender and education were associated with the use of CAM.

**Conclusions:**

Due to the high use of CAM and OTC, it is important for the prescriber to take a full history of medication use before prescribing to reduce potential problems associated with drug interactions.

## 1. Introduction

In Australia, the proportion of older people (65 years and over) is projected to increase from 13% (2.6 million people) in 2004 to 26% (6 to 6.3 million persons) by 2051 [1],[2]. The Australian Department of Health and Ageing generally defines the aged as 65 years or more [3]. More specifically, baby boomers have been defined as a person born between 1946 and 1965. There were 4.2 million people born in Australia between these years [4] (excluding baby boomers born overseas). This population group has significantly altered the structure of the population distribution in the second half of the 20^th^ century. According to the Australian Bureau of Statistics (ABS) in 2003, 27% of the population in the state of South Australia were projected to be baby boomers in 2011 [3] with 23.7% of the population of Australia baby boomers by 2014 [5]. As this group comprises a significant proportion of the population, there is the need to understand, review and optimise the health and social outcomes and, in particular, the use of medications.

A review of the literature indicates that the use of prescription medicines among older people is high, with prevalence rates ranging from 70% to 90% [6]–[11]. The rate of use is increasing, as is the mean number of prescription medicines being taken. In terms of OTC medicines, there are only limited studies and the results are impacted by using different definitions and medicines switching from prescription to OTC. The prevalence of the use of the OTC medicines among adults aged more than 65 years ranged from 31% to 96% between 1981 and 2008 in the United States of America [12]–[16]. In Australia, the prevalence of the use of OTC medicines in older people was 12.8% (1992–1993), 16.6% (1994–1995), 10.0% (2000–2001), and 17.0% (2003–2004) [17], with the overall use lower compared to the United States. In addition, it has been shown that up to two thirds of Australians use CAM [18]. For those Australians aged 65 and older, the prevalence of use was 8.6% in 1992–1993, 14.4% in 1994–1995, 9.0% in 2000–2001, and 24.2% in 2003–2004 [17]. The sale of CAM in Australia was estimated to be worth A$1.2 billion per year in 2008 [19] and in 2011, the Australian Department of Health and Ageing estimated the market growth of complementary medicines to be between 3% and 12% [19]. While CAM use is lower in older populations, the overall trend is increasing [19].

One issue related to any medicine use is polypharmacy. Polypharmacy has, however, been compromised by the use of inconsistent definitions. Polypharmacy refers to the concurrent use of medications. It is often described as taking two or more medicines concurrently over a period of time [11],[20]. The definitions of polypharmacy vary from country to country and there is still no consensus definition. In Australia, polypharmacy is usually defined as five or more drugs including prescribed, OTC medicines, and CAM [21], however, OTC medicines and CAM have not always been included in the definition and study of polypharmacy. In this study, polypharmacy refers to taking five or more medications [21] and for the purposes of this study, major polypharmacy refers to taking ten or more medications. Both definitions include prescription medicines, OTC medicines and CAM.

Details on the combination of the medicines, including prescription medicines, OTC medicines and CAM used by baby boomers and older people is still not fully understood [22]. In addition, little is known about generational differences in medicine use, particularly with regard to CAM and OTC. An assessment of the use of medicines for baby boomers and older people is important due to the increased life expectancy rates, and thus potentially longer periods of medication use, potential medication interactions and changing medication use over time.

This study aimed to examine patterns of use of medicines including prescription medicines, OTC medicines and CAM between older generations, specifically baby boomers and older people, and compare the differences between these two groups. A further investigation of the links between sociodemographic factors and medicines use are also performed. Understanding which medications are used and the differences in medicine usage among these older generations will help inform research and health policy to better meet the health care needs of the future ageing population.

## 2. Methods

This study used data from the South Australian Health Omnibus Survey (HOS), which was a representative population face-to-face survey conducted in SA annually or biannually between 1991 and 2017. HOS was a “user-pay” survey in which multiple users collected population health data. The operation of this survey was overseen by Population Research and Outcome Studies (PROS), University of Adelaide in conjunction with Harrison Health Research. Harrison Health Research was an accredited organization of Interviewer Quality Control Australia (IQCA). The methodology has been reported previously but is briefly described below [23].

### 2.1. Study population

In each survey, approximately 3000 interviews were conducted with participants aged 15 years and over. The person selected to participate was the person with the last birthday (if there is more than one adult in the household). No substitute interviews were undertaken and only one interview was conducted in each household.

### 2.2. Study setting

The HOS was conducted in South Australia with participants those living in Metropolitan Adelaide (the state capital) and rural South Australia.

### 2.3. Sampling procedure

The HOS utilised a clustered, random, multi-stage and systematic sample. Households were randomly selected based on ABS collector districts (CD). Only towns with over 1000 people were included in the sample selection process for the rural component. Hotels, hospitals, motels, hostels and other institutions were excluded from the sample. CDs were selected based on a randomly selected skip interval and the number of dwellings in the CD. Households within each of the selected CDs were then randomly selected based on a fixed skip interval from a random starting point.

### 2.4. Participant recruitment

Letters were sent to each household informing them that they had been selected for interview. If households did not indicate that they wished to be excluded from the survey, a trained interviewer visited the home to conduct the interview.

The data used in this research are the 2004 and 2008 surveys undertaken in Autumn (March-May). Overall there were n = 3,015 respondents in 2004 (response rate 65.9%) and n = 3,034 respondents in 2008 (response rate 62.8%). All future references to Health Omnibus Survey will be HOS 1 (2004 survey) and HOS 2 (2008 survey).

Ethics approvals for the HOS were obtained from the South Australian Department of Health Human Research Ethics Committee and all participants provided informed consent prior to proceeding with the face-to-face questionnaire.

### 2.5. Variables

Standard demographic information, (age, sex, household size, education, income, marital status, area of residence, work status and country of birth) were all collected. In 2004 and 2008, questions were also asked in relation to medication use. The specific questions are detailed below:

1. Prescription medicine use in both HOS surveys, participants were asked: “Are you currently using any medicines prescribed by a General Practitioner (GP) or any other health professional? This includes tablets, creams, sprays, patches etc. (but excluding the contraceptive pill)”. Participants who answered “yes” to this question were given a separate sheet to record all the medicines taken.

2. OTC medicines or CAM use. In HOS 1, the participants were asked: “Over the last year, which of these complementary or alternative medicines or health products have you used (excluding calcium, iron supplements or vitamins prescribed by your doctor)? 1. Herbal medicines; 2. Vitamins; 3. Mineral supplements; 4. Chinese medicines; 5. Homeopathic medicines; 6. Soy products; 7. Aromatherapy oils; 8. Others; 9. None”. These groupings were based on those used by the Therapeutic Goods Administration of Australia [24]. The answers were recoded into taking at least one or none. The respondent wrote the brand and name of the three main products that they used.

More detailed information was available from HOS 2, with the participants asked: “Are you currently using any non-prescribed complementary medicines? These include vitamins, fish oil, St John's Wort, and Glucosamine?” The respondents were given a sheet to write down the brand and name of the products used.

### 2.6. Weighting

All data were weighted by age, sex, area of residence and probability of selection in the household to most recent census or estimated resident population obtained from the ABS, in order to provide estimates representative of the South Australian population and to correct for sample bias. All analyses are undertaken with weighted data.

### 2.7. Analysis

Descriptive characteristics (frequencies and means) were determined using SPSS Version 20. A chi-square (χ2) test was used to determine whether there was a relationship between each age group and the use of medicines. A one-way between subject's ANOVA was conducted to compare the mean number of prescription medicines used by the three age groups. Post hoc comparisons were undertaken using the Tukey test. After determining the prevalence of the use of medicines and the mean number of medicines for baby boomers and older people, examination of the associations between the medicines used (prescription medicines, OTC medicines, CAM) and other variables of interest (independent variables including the demographic characteristics) was examined using logistic regression (backward stepwise). Variables with p < 0.25 were entered in the model [25] and after each model was formulated, the variables with p > 0.05 were subsequently removed. The Hosmer-Lemeshow test was used to determine the goodness of fit for the final logistic regression model. This test is chi-square based with a large value of chi-square (with small p < 0.05) indicating a poor fit and small chi-squared values (with a larger p-value closer to 1) indicating a good fit for the model.

## 3. Results

In HOS 1, a total of 1,818 people were classified as baby boomers (born between 1946 and 1965) and older people (born before 1946). However, it has been shown that the social and economic characteristics differ between younger and older baby boomers [26]. Thus, for the purposes of this study the baby boomer cohort was divided into a younger (1956–1965) and older (1946–1955) group [27]. There was n = 567 young baby boomers, n = 494 old baby boomers and n = 757 older people. In HOS 2 a total of 1,648 baby boomers and older people were interviewed. There was n = 538 young baby boomers, n = 463 baby boomers and n = 647 older people.

### 3.1. Prevalence of medicine use

The mean number of prescription medicines used by each group was determined. The use of prescription medication use increased with age. In 2004, there were 0.66 (SD 1.42), 1.74 (SD 2.39) and 3.11 (SD 2.59) medications used by young baby boomers compared to old baby boomers and older people respectively, with a statistically significant difference between the groups (F_(2,1794)_ = 195.89, p < 0.001). In 2008, the mean number of medications was 1.04 (SD 1.56), 1.73 (SD 2.16) and 2.84 (SD 2.43) for young baby boomers, old baby boomers and older people respectively, with a statistically significant difference between the groups (F_(2,1794)_ = 195.89, p < 0.001).

Overall, 31.7% (95% CI 28.0–35.7) of young baby boomers, 61.8% (95% CI 57.4–66.0) of old baby boomers and 84.8% (95% CI 82.1–87.2) of older people used prescription medicine in 2004. There were also differences in the use of CAM between each group (Table 1).

In 2008, the prevalence of the use of prescription medicine was 48.9% (95% CI 44.7–53.1) for young baby boomers and 68.5% (95% CI 64.2–72.6) for old baby boomers. Older people were the highest users of prescription medicines 84.9%, (95% CI 82.0–87.5, p < 0.001) (Table 2).

The prevalence of the use of polypharmacy is also shown in Table 2 and was 3.6% (95% CI 2.3–5.6), 9.5% (95% CI 7.1–12.5) and 22.3% (95% CI 19.2–25.7) for young baby boomers, old baby boomers and older people respectively (Table 2). Only a small number (less than 2%) were taking ten or more prescription medicines (major polypharmacy) for each age group.

### 3.2. Multivariable analysis

Table 3 demonstrates the demographic characteristics of those taking prescription medication and CAM in 2004. Female young baby boomers with lower levels of education were more likely to take prescription medicine and female old baby boomers who were not employed were more likely to take prescription medicine. For all three age groups taking CAM was consistently associated with sex, education and income.

Table 4 shows that prescription medicine use was associated with work status and country of birth for each age group in 2008. In addition, those who were not employed or those people who did not disclose their income were more likely to take OTC medicines. The demographic characteristics associated with taking CAM in 2008 were being female and having a higher level of education across all three age groups. The characteristics associated with young baby boomers undertaking polypharmacy were: not employed (3.40, 95% CI 1.99–5.81) and with post-secondary education (OR 2.11, 95% CI 1.22–3.64). Not being employed was also a predictor for polypharmacy among old baby boomers (OR 2.27; 95% CI 1.41–3.66), and older people (OR 2.67; 95% CI: 1.50–4.77) (Table 4).

**Table 1.** Prevalence of medicines use, HOS 1.

	Young baby boomers			Old baby boomers			Older people			
	n	%	(95% CI)	n	%	(95% CI)	n	%	(95% CI)	*p*
Taking prescription medicine										
No	382	68.3	(64.3–72.0)	186	38.2	(34.0–42.6)	114	15.2	(12.8 – 17.9)	
Yes	177	31.7	(28.0–35.7)	301	61.8	(57.4–66.0)	637	84.8	(82.1–87.2)	<0.001
Total^a^	560	100.0		488	100.0		750	100.0		
Taking CAM										
No	234	41.2	(37.2–45.3)	227	46.0	(41.6–50.4)	461	60.8	(57.3–64.2)	
Yes	333	58.8	(54.7–62.8)	266	54.0	(49.6–58.4)	297	39.2	(35.8–42.7)	<0.001
Total	567	100.0		493	100.0		758	100.0		
Taking prescription medicine and/or CAM										
No	169	30.3	(26.6–34.2)	91	18.7	(15.6–22.4)	60	8.0	(6.3–10.2)	
Yes	390	69.7	(65.8–73.4)	396	81.3	(77.6–84.4)	690	92.0	(89.8–93.7)	<0.001
Total^a^	560	100.0		488	100.0		750	100.0		

Note: a: n = 20 missing data due to incomplete information

The weighting of data can result in rounding discrepancies or totals not adding

**Table 2.** Prevalence of medicines use, HOS 2.

	Young baby boomers			Old baby boomers			Older people			
	n	%	(95% CI)	n	%	(95% CI)	n	%	(95% CI)	*p*
Taking prescription medicine										
No	275	51.1	(46.9–55.3)	146	31.5	(27.4–35.8)	98	15.1	(12.5–18.0)	
Yes	263	48.9	(44.7–53.1)	317	68.5	(64.2–72.6)	550	84.9	(82.0–87.5)	<0.001
Total	538	100		463	100		647	100		
Taking OTC medicine										
No	467	88.3	(85.3–90.8)	376	82.3	(78.6–85.6)	425	67.2	(63.4–70.7)	
Yes	62	11.7	(9.2–14.7)	81	17.7	(14.4–21.4)	208	32.8	(29.3–36.6)	<0.001
Total	529	100		457	100		633	100		
Taking CAM										
No	282	52.5	(48.2–56.6)	192	41.4	(37.0–45.9)	263	40.6	(36.9–44.5)	
Yes	256	47.5	(43.4–51.8)	271	58.6	(54.1–63.0)	384	59.4	(55.5–63.1)	<0.001
Total	538	100		463	100		647	100		
Taking prescription medicine and/or CAM										
No	163	30.9	(27.1–35.0)	68	14.9	(12.0–18.5)	53	8.3	(6.4–10.8)	
Yes	365	69.1	(65.0–72.9)	389	85.1	(81.5–88.0)	579	91.7	(89.2–93.6)	<0.001
Total	528	100		457	100		632	100		
Taking prescription medicine, OTC medicine and/or CAM										
No	155	29.4	(25.7–33.4)	67	14.7	(11.8–18.3)	48	7.6	(5.8–10.0)	
Yes	373	70.6	(66.6–74.3)	390	85.3	(81.7–88.2)	584	92.4	(90.0–94.2)	<0.001
Total^a^	528	100		457	100		632	100		
Taking 5 or more prescription medicines										
No	509	96.4	(94.4–97.7)	414	90.5	(87.5–92.9)	492	77.7	(74.3–80.8)	
Yes	19	3.6	(2.3–5.6)	43	9.5	(7.1–12.5)	141	22.3	(19.2–25.7)	<0.001
Total^a^	528	100		457	100		632	100		
Taking 5 or more prescription medicines, OTC medicines and/or CAM										
No	442	83.6	(80.2–86.5)	334	73.0	(68.7–76.8)	362	57.2	(53.3–61.0)	
Yes	87	16.4	(13.5–19.8)	124	27.0	(23.2–31.3)	271	42.8	(39.0–46.7)	<0.001
Total	528	100		457	100		632	100		
Taking 10 or more prescription medicines										
No	527	99.8	(98.9–100)	452	98.9	(97.4–99.5)	623	98.5	(97.2–99.2)	
Yes	1	0.2	(0–1.1)	5	1.1	(0.5–2.6)	10	1.5	(0.8–2.8)	0.068
Total^a^	528	100		457	100		633	100		
Taking 10 or more prescription medicines, OTC medicines and/or CAM										
No	515	97.4	(95.7–98.5)	443	97.0	(95.0–98.2)	593	93.8	(91.7–95.5)	
Yes	14	2.6	(1.5–4.3)	14	3.0	(1.8–5.0)	39	6.2	(4.5–8.3)	0.003
Total^b^	528	100		457	100		632	100		

Note: ^a^: n = 29 ^b^ n = 30 missing data due to incomplete information

The weighting of data can result in rounding discrepancies or totals not adding

**Table 3.** Multivariable analysis of demographic characteristics associated with taking prescription medicine and CAM, HOS 1.

	Young baby boomers			Old baby boomers			Older people		
	OR	(95% OR)	p	OR	(95% OR)	p	OR	(95% OR)	p
Prescription medicine									
Gender									
Male	1.00			1.00					
Female	1.90	(1.31–2.75)	0.001	2.49	(1.58–3.95)	<0.001			
Education									
No post-secondary	1.00								
Post-secondary	0.64	(0.44–0.92)	0.017						
Work Status									
Work full time/part time				1.00			1.00		
Not employed				2.25	(1.54–3.30)	<0.001	2.62	(1.64–4.18)	<0.001
Country of Birth									
Australia							1.00		
UK/Ireland							0.58	(0.36–0.94)	0.027
Other							0.54	(0.32–0.91)	0.021
CAM									
Gender									
Male	1.00			1.00			1.00		
Female	2.04	(1.44–2.90)	<0.001	3.33	(2.25–4.92)	<0.001	1.73	(1.26–2.38)	0.001
Education									
No post-secondary	1.00			1.00			1.00		
Post-secondary	1.45	(1.01–2.09)	0.044	1.81	(1.22–2.68)	0.003	1.42	(1.03–1.97)	0.035
Income									
≤ $30,000	1.00			1.00			1.00		
>$30,000	1.62	(1.02–2.58)	0.042	1.65	(1.06–2.56)	0.027	2.05	(1.42–2.95)	<0.001
Not stated	0.90	(0.38–2.14)	0.818	1.96	(0.92–4.17)	0.082	0.90	(0.55–1.50)	0.695

Note: Prescription medicines, Hosmer and Lemeshow goodness of fit test (young baby boomers: χ^2^ = 1.93, df = 2, p = 0.382, old baby boomers: χ^2^ = 0.001, df = 2, p = 1.00, older people: χ^2^ = 0.097, df = 3, p = 0.992). CAM Hosmer and Lemeshow goodness of fit test (young baby boomers: χ^2^ = 1.682, df = 5, p = 0.891, old baby boomers: χ^2^ = 1.48, df = 6, p = 0.961, older people: χ^2^ = 2.878, df = 6, p = 0.824.)

**Table 4.** Multivariable analysis of demographic characteristics associated with taking prescription medicine, OTC medicine, CAM and polypharmacy, HOS 2.

	Young baby boomers			Old baby boomers			Older people		
	OR	(95% OR)	p	OR	(95% OR)	p	OR	(95% OR)	p
Prescription medicine									
Income									
≤$30,000				1.00					
>$30,000				0.54	(0.29–0.99)	0.049			
Work Status									
Work full time/part time	1.00			1.00			1.00		
Not employed	3.03	(1.91–4.81)	<0.001	1.86	(1.17–2.97)	0.009	2.93	(1.69–5.07)	<0.001
Country of Birth									
Australia	1.00			1.00			1.00		
UK/Ireland	0.85	(0.51–1.43)	0.425	1.09	(0.62–1.94)	0.764	1.19	(0.62–2.29)	0.593
Other	0.43	(0.24–0.76)	0.004	0.52	(0.30–0.89)	0.017	0.52	(0.31–0.88)	0.014
OTC medicine									
Gender									
Male							1.00		
Female							0.69	(0.48–0.98)	0.040
Work Status									
Work full time/part time	1.00						1.00		
Not employed	2.18	(1.22–3.90)	0.009				4.14	(1.95–8.81)	<0.001
Income									
≤$30,000				1.00			1.00		
>$30,000				0.62	(0.35–1.08)	0.091	0.79	(0.50–1.23)	0.295
Not stated				0.33	(0.13–0.86)	0.023	1.61	(1.04–2.48)	0.033
Country of Birth									
Australia							1.00		
UK/Ireland							0.89	(0.56–1.41)	0.621
Other							0.52	(0.32–0.85)	0.009
CAM									
Gender									
Male	1.00			1.00			1.00		
Female	2.39	(1.67–3.42)	<0.001	2.83	(1.89–4.25)	<0.001	2.52	(1.77–3.60)	<0.001
Household size									
Single				1.00					
Living with others				0.42	(0.19–0.91)	0.028			
Education									
No post-secondary	1.00			1.00			1.00		
Post-secondary	1.65	(1.14–2.41)	0.009	1.76	(1.17–2.64)	0.007	1.61	(1.12–2.31)	0.010
Marital status									
Married/de facto				1.00					
Separated/divorced				0.41	(0.21–0.77)	0.006			
Widowed				0.46	(0.13–1.61)	0.225			
Never married				0.19	(0.06–0.56)	0.003			
Polypharmacy									
Gender									
Male							1.00		
Female							1.41	(1.01–1.95)	0.042
Work Status									
Work full time/part time	1.00			1.00			1.00		
Not employed	3.40	(1.99–5.81)	<0.001	2.27	(1.41–3.66)	0.001	2.67	(1.50–4.77)	0.001
Income									
≤$30,000				1.00					
>$30,000				0.78	(0.45–1.36)	0.388			
Not stated				0.39	(0.18–0.85)	0.017			
Education									
No post-secondary	1.00								
Post-secondary	2.11	(1.22–3.64)	0.007						

Note: Prescription medicine, Hosmer and Lemeshow goodness of fit test (young baby boomers: χ^2^ = 0.494, df = 3, p = 0.920,old baby boomers: χ^2^ = 4.92, df = 6, p = 0.554, older people: χ^2^ = 1.06, df = 2, p = 0.588)

OTC, Hosmer and Lemeshow goodness of fit test (older people: χ^2^ = 3.794, df = 7, p = 0.803)

CAM, Hosmer and Lemeshow goodness of fit test (young baby boomers: χ^2^ = 1.126, df = 2, p = 0.569, old baby boomers: χ^2^ = 1.521, df = 6, p = 0.958, older people: χ^2^ = 0.006, df = 2, p = 0.997)

Polypharmancy, Hosmer and Lemeshow goodness of fit test (young baby boomers: χ^2^ = 0.494, df = 2, p = 0.781, old baby boomers: χ^2^ = 13.926, df = 4, p = 0. 008, older people: χ^2^ = 3.472, df = 2, p = 0.176)

## 4. Discussion

This study demonstrates that the use of OTC and CAM occurs with prescription medication use and that polypharmacy, including all of these medications, occurs in over 40% of older people. The prevalence estimates for prescription medicines use for older people (approximately 85%) compares to other surveys with ranges between 87% and 90% commonly reported [10],[12],[28]–[32], although lower estimates have been reported from USA (81%) [6]. A comparable Australian study conducted in 2009 and 2010 [28] indicated 87.1% of older people (65 years and over) used prescription medicines, which is in line with our estimates.

Previous studies [33]–[34] have shown conflicting results regarding whether OTC medicine use increases with age, but in this study, the use of OTC medicines clearly increased with age, with estimates of OTC medicines use being 11.7%, 17.7% and 32.8% for young baby boomers, old baby boomers and older people respectively in HOS 2. These prevalence estimates were lower when compared to surveys conducted in USA [6],[13],[33]–[34] with prevalence estimates ranging from 42% to 90%. The discrepancies can be explained by differences in the definition of OTC medicines and differences in subsidised accessibility to prescription medicines. Alternatively, the reported use of OTC medicines for older people (65+ years) in this study (32.8% HOS 2) was higher compared to a previous Australian study, the Australian Longitudinal Study of Ageing (ALSA) [17] which reported a prevalence of 17.7% in 2003–2004. This difference in estimates could potentially be a real increase although ALSA did not classify some medicines, (for example salbutamol inhalers) as OTC medicines, while in HOS 2 salbutamol inhalers were classified as OTC medicines. This again highlights the difference in use of standard definitions in this research area, and the need for a consistent approach.

Conflicting results were also seen regarding the use of CAM, with the use decreasing with age in HOS 1 but increasing with age in HOS 2. The use of CAM increased in older people between HOS 1 (39.2%) and in HOS 2 (59.4%) but decreased use was found in young baby boomers from 58.8% in HOS 1 to 47.5% in HOS 2. These findings raise the question whether CAM use did chang over time in these age groups and a more recent study assessing the use of CAM in baby boomers and older people is required to add clarification to these results. This is important, as the increased use of CAM in older people could potentially increase medicines interactions and adverse reactions. A national population-based survey of Australian people aged over 50 years, conducted in May-June 2005, indicated the prevalence of the use of CAM was 57.8% [28] and another specific study produced estimates of 60.8% for CAM use in SA older people [3]. Again, the prevalence of CAM was slightly higher compared to HOS 1 and HOS 2 and this may be due to the type of CAM included in the national survey with, for example, acupuncture also included in their definition of CAM. ALSA reported the prevalence of the CAM use for older people to be 24.4%. This prevalence was lower than the HOS studies possibly due to the questions being asked in ALSA regarding all medicines used in the past two weeks, instead of the past year as asked in HOS.

The use of CAM in Australia is influenced by media reports. In 2007, Arthritis Australia indicated that fish oil had shown evidence for the relief of rheumatoid arthritis pain [35]. In 2008, the National Heart Foundation released a statement recommending fish oil capsules or liquid to lower the risk of coronary heart disease [36]. Whether these announcements have increased the use of fish oil in the Australian population has yet to be formally determined. However, a study has shown that the Australian Broadcasting Corporation's science journalism program Catalyst program has changed statin use in Australia. The program, which aired in 2013, resulted in a 28.8% decrease in statin continuation [37] in the first week. The results of this study may have been influenced by the Arthritis Australia and National Heart Foundation announcements (as the timeframe of the data collection overlapped with the media reports) and could have association with the use of CAM. The inappropriate use of CAM could significantly impact on the health outcomes of older people and their use in the ageing population is significant [38]–[39].

Moreover, many people are unlikely to disclose their use of CAM to their conventional medical practitioner [40]–[43], with a study showing that as many as 70% of people did not disclose their use of CAM [44]. This contributes to difficulties for health care teams monitoring patients' health outcomes due to the interaction between medicines and natural products and adverse reactions from CAM. This is especially true for older people whose hepatic clearance is deteriorating, which could result in a toxicity of CAM in the body. Nevertheless, CAM use has been shown to be associated with higher health status in ageing populations [45] and the users have been shown to be satisfied with CAM interventions [45].

The presence of marketing forces [46], accessibility to information, existing chronic conditions (i.e. pain, diabetes, cardiovascular disease) and the dissatisfaction of conventional medicine have all been proposed as explanations for the increasing popularity of CAM use [47]. Baby boomers with their higher educational background compared to older people may seek new approaches to address their health needs and expectation. A growing self-empowerment of their own medicines management of their chronic conditions could possibly increase the use of CAM in this generation.

Baby boomer rates of chronic disease are expected to increase exponentially. In all likelihood, baby boomers could further increase their CAM use as their chronic disease profile increases. If this is the case the polypharmacy will also increase. Other important considerations could be the potential for additional adverse reactions associated with medicines use, medicines errors, drug interactions, and less than optimal compliance with medicines regimes, which could all result in additional hospitalisation and increased health expenses.

### 4.1. Strengths and limitations

Limitations of this study are that despite its inherent value, the cross-sectional design of the data analysed does not show cause and effect. Additionally, as the data are self-reported, there may have been recall bias among participants. Also, the sampling frame excluded people who resided residential care facilities and these groups are known to have high use of medicines. These analyses are descriptive and as such inform the understanding of differences in medication use between baby boomers and older people with respect to socio-demographic characteristics, chronic diseases, health risk factors and health outcomes. Large, population-based longitudinal studies involving baby boomers and older people are needed in the future.

The strengths of the study are its large, state representative sample of baby boomer and older people, and the inclusion of a wide range of demographic characteristics that could be evaluated against medication use. There have been limited studies that have examined the combination of prescription medicines, OTC medicines and CAM use in the population. There are also relatively few studies that have assessed changes in use over time in the population and linked the use of medication to health outcomes in the population. The comprehensive literature review highlights the gaps in the literature although there were limited publications solely on baby boomers. Confounders for baby boomers and older people are not known specifically.

## 5. Conclusion

This research has provided additional information and insight into the use of medicines and health outcomes and adds depth to existing knowledge in this domain. Monitoring of medicine-related problems, including the adverse reactions associated with some CAM, long-term effects, ethical implications and overall inappropriate use of medicines is recommended due to the high prevalence and association with polypharmacy. Guidelines need to be promoted highlighting the need for GPs to routinely discuss the risk and benefits of specific CAM approaches with their patients, to promote the safe use of medicines and effective treatment for chronic conditions. Additional education of the public regarding appropriate medicines use is warranted.

The cost implications associated with the high prevalence rates are substantial and requires an overhaul of the Australia health care system in terms of access to medicines use, prescription use, and GP management of medicines to meet the need of older Australians in the near future. A model utilising professions uniquely placed to identify the drug interactions and adverse effects in light of an increased use of prescription medicines and OTC medicines is important to ensure that quality of life is maintained where possible, and better health outcomes are achieved.
